# Panel Comparative Analysis Tool: An Open-Source Comparative Analysis Tool for Next-Generation Sequencing Panel Target Regions

**DOI:** 10.1016/j.jmoldx.2024.01.015

**Published:** 2024-03-18

**Authors:** André Oszwald, Lucia Zisser, Eva Compérat, Leonhard Müllauer

**Affiliations:** ∗ Department of Pathology, Medical University of Vienna, Vienna, Austria; † Division of Nuclear Medicine, Department of Biomedical Imaging and Image-Guided Therapy, Medical University of Vienna, Vienna, Austria

## Abstract

Multigene next-generation sequencing (NGS) panels have become a routine diagnostic method in the contemporary practice of personalized medicine. To avoid inadequate test choice or interpretation, a detailed understanding of the precise panel target regions is required. However, the necessary bioinformatic expertise is not always available, and publicly accessible and easily interpretable analyses of target regions are scarce. To address this critical knowledge gap, we present the Panel Comparative Analysis Tool (PanelCAT), an open-source application to analyze, visualize, and compare NGS panel DNA target regions. PanelCAT uses Reference Sequence, ClinVar, and Catalogue of Somatic Mutations in Cancer mutation census databases to quantify the exon and mutation coverage of target regions and provides interactive graphical representations and search functions to inspect the results. We demonstrate the utility of PanelCAT by analyzing two large NGS panels (TruSight Oncology 500 and Human Pan Cancer Panel) to validate the advertised target genes, quantify targeted exons and mutations, and identify differences between panels. PanelCAT will enable institutions and researchers to catalog and visualize NGS panel target regions independent of the manufacturer, promote transparency of panel limitations, and share this information with employees and requisitioners.

## Key Points

• PanelCAT enables automatic analysis of DNA target regions of NGS panels from BED files, including optional mask files.
• PanelCAT uses RefSeq, ClinVar, and COSMIC Cancer Mutation Census databases to determine protein-coding base and variant/mutation coverage.
• Interactive visualization allows intuitive assessment and comparison of panel coverage and saving of graphs as images.
• Tables with search function allow researchers to assess specific exons/mutations of interest and save filtered data as text.

Precision oncology routinely involves next-generation sequencing (NGS) of tumor DNA to identify therapeutically actionable targets or diagnostically relevant mutations that critically direct patient management.^1^ Most multigene sequencing panels do not cover entire genes, but only variable portions of genes that are considered most relevant (ie, predominantly protein-coding sequences and tumor mutational hot spots). Therefore, both the choice of an adequate test and its interpretation, especially regarding the certainty of negative findings, crucially depend on detailed knowledge of the portions of genes and genetic alterations that are assessed by a panel.

Target regions of commercial NGS panels are typically specified in a panel-specific Browser Extensible Data (BED) file by a list of chromosome numbers and start and stop coordinates.^2^ Although this information is an essential part of the test documentation, it is not useful to understand panel target regions in detail without further analysis for several reasons: it does not inform on the nontargeted portions of genes without comparison to a reference genome; the provided information on target genes, transcripts, and exons is not updated alongside the transcript databases [eg, Reference Sequence (RefSeq^3^)]; the target regions must be systematically compared with mutation/variant databases to determine pathogenic mutations that can be detected; and, last, genomic positions with known high rates of erroneous variant calls are often masked during secondary analysis, but these positions are defined in separate files.

To our knowledge, there is no current application that integrates these various sources into high-level, summarized analyses and visualization of NGS panel target regions. Consequently, the lack of detailed publicly available data on precise panel targets, and the barriers to generate it because of the required bioinformatic expertise, portends the risk of inadequate test choice and test misinterpretation. To reduce this risk, we developed the Panel Comparative Analysis Tool (PanelCAT), an application that allows researchers to analyze, visualize, and compare DNA target regions of NGS panels within a user-friendly interface, and provides a platform to clearly communicate this information to others. The use of this tool is demonstrated by analyzing two large multigene NGS panels, the TruSight Oncology 500 (TSO500; Illumina, Hayward, CA) and the Human Pan Cancer Panel (QPC; Qiagen, Hilden, Germany), to provide a more detailed documentation of their targeted genes, exons, known pathogenic mutations, and differences between the panels, than has been available to date.

## Materials and Methods

### PanelCAT Code

PanelCAT code was generated, and all analyses were performed, in R version 4.3.0 (*https://www.r-project.org*) within RStudio version 2023.03.0 (*https://posit.co/download/rstudio-desktop*). Genomic ranges (target regions and variant coordinates) were analyzed using the GenomicFeatures^4^ package. Graphs were drawn using ggplot2^5^ and plotly^6^ packages. A browser-based implementation of the script was generated using ShinyR (*https://github.com/rstudio/shiny*). PanelCAT is provided under the open-source license AGPLv3, and the source code is available (*https://github.com/aoszwald/panelcat*, last accessed November 11, 2023). The basic procedure of the panel analysis is outlined in the next paragraph.

PanelCAT accepts target region files as input (containing columns for chromosome and start and end position of target regions), and optionally the mask region file (also containing chromosome and start and end coordinates). The application first determines the intersection between the panel target regions and RefSeq^3^ exon coordinates to systematically identify target genes, and subsequently all exon ranges of target genes. The exon ranges of each targeted gene are then intersected with the panel target regions to quantify the targeted portion of protein-coding bases per gene. Targeted mutations are then identified by intersection of the panel target regions with the coordinates of mutations in the ClinVar^7^ and Catalogue of Somatic Mutations in Cancer (COSMIC)^8^ databases. Optionally, the mask file is incorporated in the analysis to identify and determine the portion of masked bases and mutations. The summarized output data are combined into lists of items and saved as R data objects. Panels that were previously analyzed and saved in this form are preloaded the next time the application is started. The panel analysis output can also be used for analysis outside of PanelCAT; within R, the panel data and listed subitems can be accessed via the $ operator. During this study, individual data were accessed in this way and further processed in R independently of the PanelCAT functions to identify discrepancies between the advertised gene list and the confirmed gene list.

### Data Sources

BED files indicating target regions of NGS panels and corresponding mask files, including Illumina TSO500 and Qiagen Pan-Cancer Panels, were obtained from the customer support of the manufacturers, or obtained while using a product. The TSO500 mask file was provided by Illumina. These files are not provided as part of PanelCAT.

ClinVar (*https://www.ncbi.nlm.nih.gov/clinvar*, last accessed May 23, 2023) and RefSeq (GRCh37, *https://www.ncbi.nlm.nih.gov/refseq*, last accessed May 23, 2023) databases are not provided as part of the software download, but will automatically be obtained by PanelCAT from the National Center for Biotechnology Information FTP server. The COSMIC cancer mutation census data version 98 (*https://cancer.sanger.ac.uk/cosmic*, last accessed May 25, 2023) must be downloaded manually (because they are accessible only after online registration), as outlined by the instructions in the GitHub repository (*https://github.com/aoszwald/panelcat*).

## Results

### General Function of PanelCAT

PanelCAT (*https://github.com/aoszwald/panelcat* and *http://panelcat.net*, last accessed November 11, 2023) provides functions to automatically distill descriptive information from panel target region files and public databases, and to display these data to facilitate evaluation and comparison of panels. To analyze a panel, PanelCAT is provided with the target region file (typically with a .bed file suffix, but others may be acceptable), and optionally a mask file (indicating regions where variant calls are unreliable and will be filtered out). The application then determines the overlap between target regions and protein-coding bases per gene in RefSeq,^3^ thereby identifying the target genes, and subsequently, the overlap between target regions and known pathogenic and likely pathogenic mutations in ClinVar^7^ and tier 1 to 3 oncogenic mutations in COSMIC^8^ cancer mutation census (CMC) databases. The output data are saved in a compact form that can be used in PanelCAT or explored independently in R Statistics.

**Figure 1:**
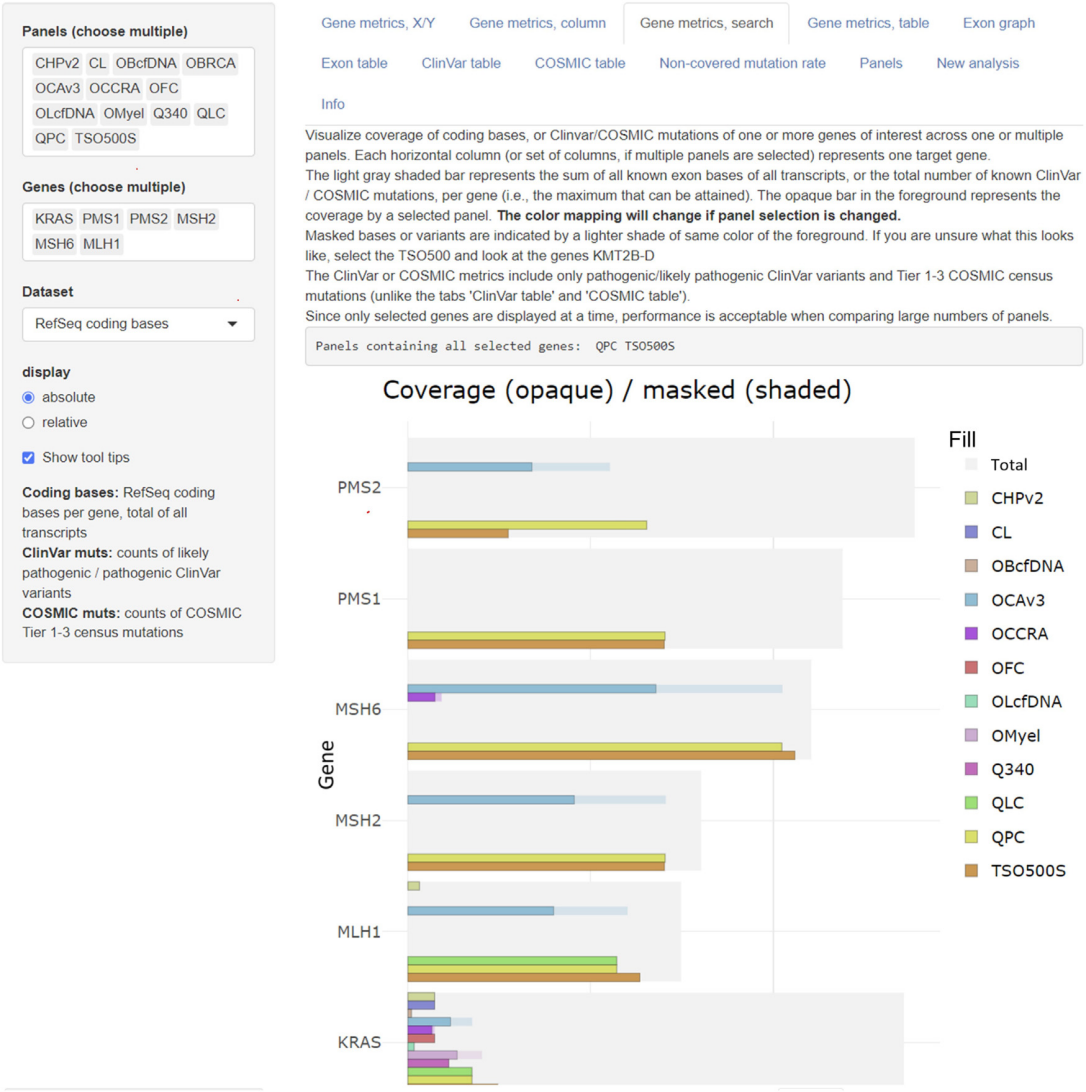
Exemplary Panel Comparative Analysis Tool (PanelCAT) user interface and visualization options. Among other functions (see Supplemental Figure S1), PanelCAT allows researchers to visualize coverage of multiple genes of interest across multiple panels. The genes *PMS1*, *PMS2*, *MSH2*, *MSH6*, *MLH1*, and *KRAS* were searched across multiple previously analyzed panels (including several other panels from Thermo Fisher, Waltham, MA; and Qiagen). Only the TruSight Oncology 500 (TSO500S) and the Human Pan Cancer (QPC) panels target all the genes of interest. Gene names are not italicized in this figure because of software limitations. The method of masking variant calls is different between the Illumina TSO500 and Thermo Fisher panels: in the TSO500, variant calls in masked positions are filtered absolutely, whereas in Thermo Fisher panels, masking depends on additional base call quality metrics (not discussed in this article). Web sources: ClinVar (*https://www.ncbi.nlm.nih.gov/clinvar*, last accessed May 23, 2023), Reference Sequence genes and transcripts (GRCh37, *https://www.ncbi.nlm.nih.gov/refseq*, last accessed May 23, 2023), and Catalogue of Somatic Mutations in Cancer mutation census version 98 (*https://cancer.sanger.ac.uk/cosmic*, last accessed May 25, 2023). This figure also appears as Supplemental Figure S1C. CHPv2, Cancer Hotspot Panel version 2 (custom design); CL, Colon/Lung panel (custom design); OBcfDNA, Oncomine Breast cfDNA Assay; OCAv3, Oncomine Comprehensive Assay version 3; OCCRA, Oncomine Childhood Cancer Research Assay; OF, Oncomine Focus Assay; OLcfDNA, Oncomine Lung cfDNA Assay; Omyel, Oncomine Myeloid Research Assay; Q340, Custom Qiagen Lung Panel; QLC, Qiagen Lung Cancer Panel.

**Supplemental Figure S1:**
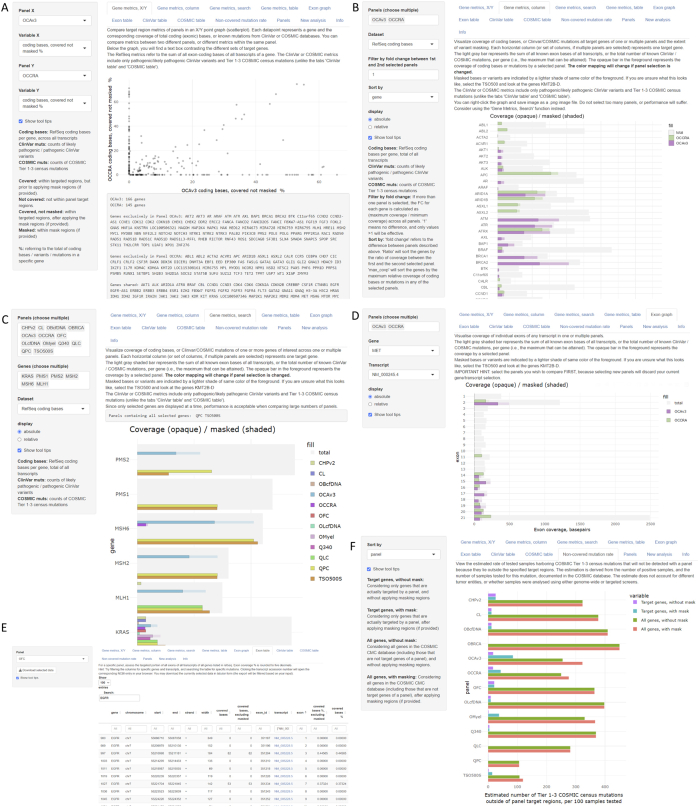
Panel Comparative Analysis Tool user interface and visualization options. Panel DNA target region coverage can be investigated in different visualizations, some of which are exemplified here. **A:** An X/Y point graph allows researchers to compare any coverage metric from any previously analyzed panel. In this case, it shows the relative coverage of nonmasked exon bases per gene [as a percentage of the total exon bases of all known Reference Sequence (RefSeq) transcripts for a gene] of the Oncomine Comprehensive Assay version 3 (OCAv3) and the Oncomine Childhood Cancer Research Assay (OCCRA; both from Thermo Fisher, Waltham, MA). **B:** A horizontal bar plot shows the absolute coverage of exon bases (opaque foreground), the masked portion (shaded foreground), and the relation to total exon bases of all known RefSeq transcripts per gene (light gray background). **C:** The genes *PMS1*, *PMS2*, *MSH2*, *MSH6*, *MLH1*, and *KRAS* were searched across multiple previously analyzed panels (including several other panels from Thermo Fisher and Qiagen). Only the TruSight Oncology 500 (TSO500) and the Human Pan Cancer (QPC) panels target all the genes of interest. **C:** Also appears as main text Figure 1. **D:** Coverage of individual exons is visualized in a horizontal column graph. In this example, the graph shows exon base coverage of the *MET* transcript NM_000245.5 by the OCAv3 and OCCRA panels. It is evident that coverage of most exons is different between panels, in particular regarding exons 11, 17, and 18, which are only targeted by either one of the panels. **E:** Detailed exon coverage metrics can be viewed and searched in a table, and the filtered data can be easily exported to a text file for later use. In this case, the table shows exon coverage metrics of the *EGFR* transcript NM_005228.5. Only the first 9 of 28 exons are displayed in this figure. **F:** The estimated frequency of Catalogue of Somatic Mutations in Cancer (COSMIC) cancer mutation census (CMC) tier 1 to 3 mutations that are not targeted by panels, given as the cumulative number of mutations expected in 100 samples, assuming that 100 samples are tested with a specific panel. The target genes columns indicate estimates for panels in relation to their specific target genes, whereas the all genes columns indicate the estimates for panels in relation to all genes (with tier 1 to 3 mutations in the COSMIC CMC database). For instance, although the Oncomine *BRCA* assay (OBRCA; Thermo Fisher) covers only a few genes (and the estimated frequency of nontargeted mutations across all genes is high), the high coverage of the explicit target genes (*BRCA1* and *BRCA2*) results in a low estimate of nontargeted mutations in the explicit target genes. Gene names are not consistently italicized in this figure because of software limitations. The method of masking variant calls is different between the Illumina TSO500 and Thermo Fisher panels: in the TSO500, variant calls in masked positions are filtered absolutely, whereas in Thermo Fisher panels, masking depends on additional base call quality metrics (not discussed in this article). Web sources: ClinVar (*https://www.ncbi.nlm.nih.gov/clinvar*, last accessed May 23, 2023), RefSeq genes and transcripts (GRCh37, *https://www.ncbi.nlm.nih.gov/refseq*, last accessed May 23, 2023), and COSMIC cancer mutation census version 98 (*https://cancer.sanger.ac.uk/cosmic*, last accessed May 25, 2023).

The application provides several useful visualization options to analyze and compare panels, briefly outlined here. In a point graph (Gene metrics, X/Y), users can contrast target coverage metrics of RefSeq, ClinVar, and COSMIC databases from all analyzed panels on the level of individual genes. This function can be used to compare between two panels (eg, to determine differences in target gene coverage) (Supplemental Figure S1A) or to compare different metrics within a single panel (eg, to evaluate the relationship between covered protein-coding bases and pathogenic mutations). A panel-wide representation of gene coverage (and masked portions) of protein-coding bases, ClinVar variants, or COSMIC mutations is also provided across multiple panels in a horizontal column plot (Gene metrics, column) (Supplemental Figure S1B). These two options are particularly useful to gain a basic overview of differences between panels. Specific genes of interest can be searched to visualize coverage across panels using a method that was optimized to display many panels simultaneously (Gene metrics, search) (Figure 1 and Supplemental Figure S1C), alongside an indication of which of the searched panels target all genes of interest. The gene-level data underlying the visualizations described in the sentence above can be queried in a customizable and searchable table (Gene metrics, table), where data can also be exported to text and downloaded for later use.

The coverage of individual exons by panel target regions is visualized in a horizontal column plot (Exon graph) (Supplemental Figure S1D), where users can choose specific entries of all transcripts of all genes, and select multiple panels for comparison. The underlying exon-level data can also be queried in a table (Exon table) (Supplemental Figure S1E) with search and export function. These visualizations are particularly useful to characterize differences in gene coverage between panels in greater detail.

The ClinVar and COSMIC database entries of mutations targeted by individual panels can be inspected in tables (COSMIC table and ClinVar table). The exon, ClinVar, and COSMIC tables can be filtered for each column independently (eg, by specific genes, transcripts, exons, coding or amino acid changes, or genome coordinates), and include hyperlinks that refer a user directly to the respective RefSeq, Clinvar, or COSMIC entry. Last, PanelCAT provides a visualization of the estimated cumulative frequency of COSMIC CMC tier 1 to 3 mutations that are not targeted by panels. The estimate is provided per panel for the actual target genes only, and for all genes, both with and without considering variant masking (Noncovered mutation rate) (Supplemental Figure S1F). In addition to the previous options, these metrics may assist users in estimating the clinical utility of panels with similar sets of target genes.

Users can access an online instance of PanelCAT (*http://panelcat.net*) or implement a local instance of PanelCAT with little effort, as outlined in the online repository (*https://github.com/aoszwald/panelcat*). When run locally, PanelCAT automatically obtains the current ClinVar (released weekly) and RefSeq databases on first use. These, along with all previously processed panels, can be updated in a single step (in the New Analysis tab); previous versions of databases and panel analyses are stored alongside current files for later reference and documentation. The COSMIC CMC database (updated every several months) requires manual download (because of required online registration) and replacement of the local file. When hosted as a network service (such as *http://panelcat.net*), analyses of target regions provided by client users are not saved permanently, but can be downloaded and re-uploaded at a later time, as described in the New Analysis tab of the application.

In summary, PanelCAT offers multiple useful and intuitive functions to substantially improve the transparency and accessibility of NGS panel target region documentation. The newly developed tool was next used to analyze two large panels currently used in both clinical and research settings; the Illumina TSO500 and the Qiagen QPC panels. Although the target regions can be requested as part of the panel documentation, explicit coverage of genes and mutations is not provided. However, informed clinical use requires detailed information, so the authors used PanelCAT to characterize their target regions in detail and explore their differences.

### Mutation Coverage in the Qiagen Pan Cancer Panel Is Similar to or Greater than in TSO500, Despite Lower Exon Coverage

In their respective product documentation, the TSO500 and QPC panels advertise the same 523-gene targets for analysis of small variants (eg, single-nucleotide variations and insertions and deletions). The advertised genes were first compared with the target genes identified using PanelCAT. The QPC target regions overlapped with exons of 603 genes, including all advertised genes. By contrast, the TSO500 target regions overlapped with exons of 625 genes, but these included only 521 of the 523 advertised targets. The two remaining target genes (*HLA-B* and *HLA-C*) do not overlap with the TSO500 target regions. Accordingly, the authors did not find any variant calls in *HLA-B* or *HLA-C* in a representative set of 400 samples analyzed with the TSO500 panel (including unfiltered variant call files in 10 samples). Alterations in genes corresponding to major histocompatibility complex class I (eg, *HLA-B* or *HLA-C*) have been postulated to promote tumor evasion of immune surveillance (eg, by restricting neoantigen presentation),^9^^,^^10^ although no guideline recommendations to test *HLA* genes exist currently.

**Figure 2:**
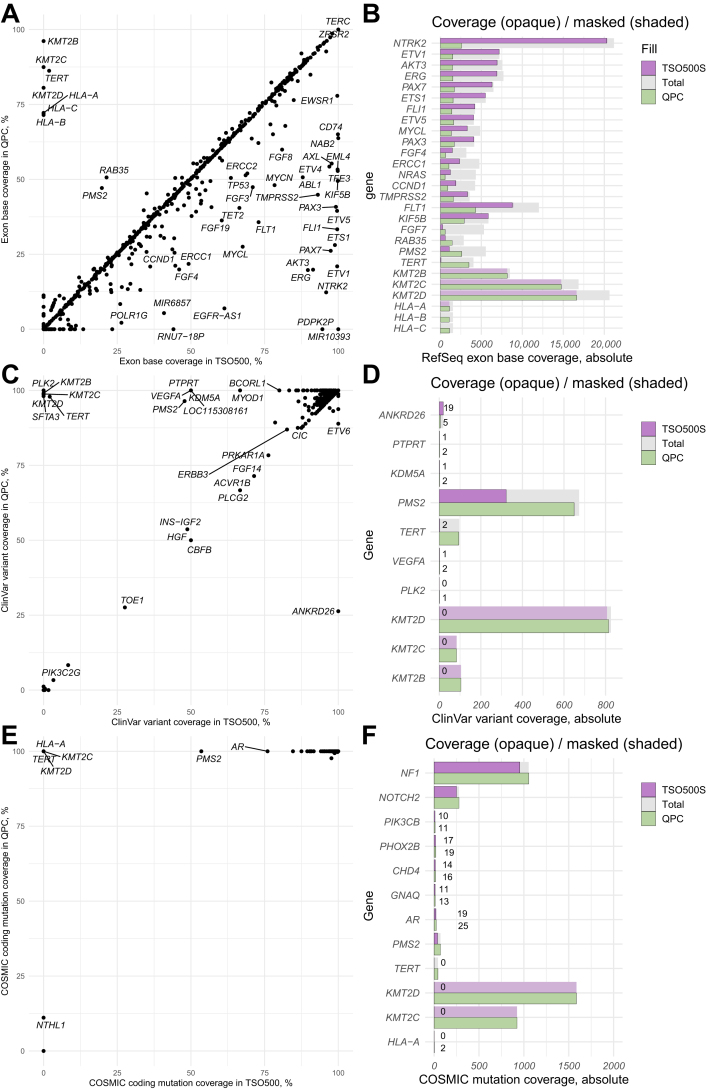
Exon base and mutation coverage in Qiagen Human Pan Cancer (QPC) and TruSight Oncology 500 (TSO500) panels. **A**–**F:** Coverage of exon bases (**A** and **B**), ClinVar variants (**C** and **D**), and Catalogue of Somatic Mutations in Cancer (COSMIC) cancer mutation census mutations (**E** and **F**) in QPC and TSO500 panels. Exon base coverage is provided as a percentage of the total known exon bases of all Reference Sequence (RefSeq) transcripts of a gene. Variant and mutation coverage is provided as the percentage of all alterations registered in ClinVar or COSMIC databases for a specific gene. **A:** It is evident that for most genes, coverage is highly correlated between TSO500 and QPC panels, with a trend toward higher exon base coverage of individual genes in the TSO500 panel. **B:** Genes with the greatest differences between panels are shown in detail. **C**–**F:** Surprisingly, mutation coverage is equal or greater in the QPC panels. **F:** In some genes (such as *KMT2C-D*), this is evidently due to extensive variant masking (shaded bars). Note that the TSO500 uses a mask file to filter variant calls at positions with high error rates (shaded purple bars), but the QPC does not. **B**, **D**, and **F:** Only genes with large relative differences are shown (filtered by fold changes of 2, 1.5, and 1.1, respectively). Web sources: ClinVar (*https://www.ncbi.nlm.nih.gov/clinvar*, last accessed May 23, 2023), RefSeq genes and transcripts (GRCh37, *https://www.ncbi.nlm.nih.gov/refseq*, last accessed May 23, 2023), COSMIC cancer mutation census version 98 (*https://cancer.sanger.ac.uk/cosmic*, last accessed May 25, 2023).

The targeted exon-coding bases of each gene were identified next. Genes with the greatest coverage were searched first. In the TSO500, 20 genes had exon coverage >95% (including *NAB2*, *TERC*, *CD74*, *TFE3*, *KIF5B*, *EML4*, *EWSR1*, *FLI1*, *ETV1*, *ETV5*, and *PAX3*, all >99%), whereas in the QPC, it was only six genes (*TERC*, *ZRSR2*, *ATR*, *POLD1*, *KMT2B*, and *RECQL4*). A strong direct correlation was found between the base coverage of TSO500 and QPC panels (Pearson *r* = 0.81, *P* < 2 × 10^–^^16^), and no significant difference was found in mean exon base coverage per gene (TSO500 versus QPC, 50.3% versus 48.4%; *P* = 0.23) (Figure 2A). Target genes where relative coverage was considerably greater in the TSO500 than in the QPC, including *NTRK2*, *ETV1*, *AKT3*, *ERG*, and *PAX7*, but only a few genes with greater coverage in the QPC panel, notably *PMS2*, *TERT*, *HLA-B*, and *HLA-C* (Figure 2B) were identified. Importantly, four targeted genes (*HLA-A*, *KMT2B*, *KMT2C*, and *KMT2D*) showed total masking of all target regions in the TSO500 panel (but not in the QPC, which does not use a mask file). No variant calls were found in these genes in a representative set of 400 samples analyzed with the TSO500 panel.

ClinVar lists approximately 50,000 known variants labeled pathogenic or likely pathogenic in the advertised TSO500 and QPC target genes. Although the TSO500 targeted 92.5% of these variants (94.8% without variant masking), the QPC targeted 97.4%, despite lower exon coverage. Consequently, targeting of all pathogenic variants was achieved for 182 genes in the TSO500 panel (200 without masking), and 223 genes in the QPC panel. In the TSO500 panel, no pathogenic variants were targeted in eight genes (of which six were due to variant masking), whereas in the QPC, it was only three. In both panels, most (QPC, 51%; TSO500, 64%) nontargeted variants occurred in two similar sets of only 10 genes, in both cases including *NF1* and the DNA repair genes *MLH1*, *MSH2*, *BRCA1*, *BRCA2*, and *ATM* (Supplemental Table S1). Most differences between panels were attributable to either greater exon coverage in the QPC (*PMS2* and *TERT*) (Supplemental Table S1) or extensive masking in the TSO500 (*KMT2B*, *KMT2C*, and *KMT2D*) (Figure 2, C and D, and Supplemental Table S2).

The COSMIC CMC database version 98 lists approximately 43,000 unique mutations occurring in genes targeted by the TSO500 and QPC panels, of which most (93.4%, or 99.5% without variant masking) are targeted by the TSO500, and all by the QPC (100%) (Figure 2, E and F). Because of masking, no mutations are targeted by the TSO500 in *HLA-A*, *KMT2C*, and *KMT2D*. Independent of masking, the QPC panel more extensively targeted mutations in *NF1*, *TERT*, and *PMS2* than the TSO500. The authors estimated the frequency of samples to harbor nontargeted mutations by calculating the positive sample proportion of unique mutations in the CMC data set, and cumulating the frequency of all nontargeted mutations. The rate of noncovered CMC tier 1 to 3 mutations in targeted genes per sample was lower in the QPC (0.005) than in the TSO500 (0.14, 0.02 without masking), suggesting that 1 in 8 (TSO500, or 1 in 50 without masking) or 1 in 200 (QPC) samples would harbor potentially oncogenic mutations (ie, tier 1 to 3 COSMIC census mutations) that cannot be detected with the panels, in one of the panel target genes.

## Discussion

Detailed knowledge of the target regions of NGS panels is important for the correct choice and interpretation of molecular tests, but it is not typically well illustrated by the test manufacturer, and usually requires bioinformatic analysis to acquire. This study introduces PanelCAT, a novel open-source tool that can be used by laboratories or NGS panel distributors to analyze NGS target regions and share this information to enable more informed decisions. PanelCAT currently does not assess fusion or copy number events detected by panels, but respective features may be implemented in the future.

PanelCAT enables rapid assessment and rich visualization of the designed target regions of NGS panels without bioinformatic expertise. As an example, we expanded in detail on the existing and incomplete documentation of two large NGS tests (Illumina TSO500 and Qiagen QPC). PanelCAT quantified precise exon coverage and identified genes with poor coverage, extensive variant masking, differences between panels, and even discrepancies to the advertised gene list. Thus, we found that unlike the QPC, the TSO500 does not target *HLA-B* and *HLA-C*; *KMT2B*, *KMT2C*, and *KMT2D* are extensively masked in the TSO500 and will not yield variant calls after filtering; and exon sequences of *PMS2* and *TERT* are substantially better covered in the QPC panel independent of variant masking. In addition, we used PanelCAT to describe the different coverage of individual exons of *PMS2* in the panels.

PanelCAT offers unique and specialized functions that distinguish it from other software with more general use. Theoretically, the target regions of an NGS panel can also be visualized in a linear manner using software, such as the Integrated Genomic Viewer,^11^ but obtaining summarized data and comparisons between large panels would be extremely tedious. To our knowledge, there is no software dedicated to the purpose of comparing NGS target regions. Some commercial NGS reagent providers offer online software tools (eg, GeneGlobe and Qiagen) to search and design panels, typically with basic feedback on the portion of the desired target regions that are covered by a produced panel design. However, the feedback does not inform of coverage of individual genes and exons, and comparisons or visualizations of panels are not supported.

PanelCAT is also different from the Panel Informativity Optimizer (PIO) method, previously demonstrated to assist in optimizing NGS panel design.^12^ Because PanelCAT does not generate new target regions, it only indirectly assists in panel design by highlighting deficits in exon or mutation coverage in particular genes of interest. Compared with PIO, PanelCAT provides superior functions to analyze and compare existing (or proposed) panels. Crucially, PIO cannot process complex target regions or conventional BED-format files, only lists of complete genes or exons. Most panels target incomplete genes or exons, and would thus be inaccurately represented using PIO. Consequently, the limited panel benchmarking functions of PIO cannot inform on precise exon coverage, whereas PanelCAT provides detailed information on the level of genes, exons, and individual mutations. In contrast to PIO, which provides a linear data pipeline from input to output, PanelCAT is a platform to collect panel analyses and visualize them for frequent inspection. In summary, PanelCAT provides opportunities that have not yet been demonstrated with previous methods, albeit with features designed more for panel end users than panel developers.

In contrast to PIO, PanelCAT does not use a variety of mutation databases to account for the heterogeneity of mutation frequencies across different disease entities. Although panels are often designed for specific disease entities or groups thereof, many widely used panels (eg, Thermo Fisher Oncomine Focus or Illumina TSO500) were designed to cover a wide range of disease entities, and we therefore also chose a disease-agnostic approach for PanelCAT. However, the variant databases used by PanelCAT can be preprocessed by users to focus the analysis entirely on mutations that are relevant in a specific disease context.

PanelCAT was designed for users with limited or no information technology support, and no more than basic computational expertise. We have aimed to make installation of the software as simple as possible and provide a guide for this process in the online repository (*https://github.com/aoszwald/panelcat*). The software functions on a local device (without installation of software besides R statistics and R Studio); however, users may also choose to analyze and visualize their panels using the online instance (*http://panelcat.net*). Similarly, PanelCAT can be hosted as a private network service with the use of ShinyServer (also outlined in the repository). PanelCAT reference databases can be easily updated, and stored panel analyses can be managed within the operating system's file system without database experience.

Although multigene NGS panels are currently the standard procedure in many institutions, routine whole-exome sequencing of tumor specimens is being increasingly performed. Because of the high performance of the underlying packages,^4^ PanelCAT could be used to analyze target regions of whole-exome panels. However, the increased rendering time of some of the implemented visualization methods could be impractical. Nevertheless, the PanelCAT output data, saved as R objects, could be used outside of PanelCAT to plot custom graphs demanding less computation.

In conclusion, PanelCAT is a powerful solution to current shortcomings in the presentation, analysis, and awareness of NGS panel target regions. This software may improve the transparency of NGS panels and facilitate more informed decisions in test choice and interpretation, thus constituting a valuable addition to the expanding repertoire of available tools.

## Disclosure Statement

None declared.
